# Sacroiliac Joint Radiofrequency Ablation Therapy After Sacroiliac Joint Fusion

**DOI:** 10.7759/cureus.91286

**Published:** 2025-08-30

**Authors:** Seung J Lee, Nwadi Igwe, Kanchana Gattu, Thelma Wright

**Affiliations:** 1 Anesthesiology, University of Maryland School of Medicine, Baltimore, USA; 2 Anesthesiology, University of Maryland Medical Center, Baltimore, USA

**Keywords:** chronic nonspecific low back pain, minimally invasive pain management, radiofrequency ablation (rfa), sacroiliac joint dysfunctional pain, sacroiliac joint fusion

## Abstract

Sacroiliac joint dysfunction (SIJD) is a significant contributor to lower back pain, and the condition often mimics other lower back pain syndromes, necessitating accurate diagnosis through history, physical examination, provocative tests, and imaging studies.

We present a 67-year-old man with a history of sarcoidosis, deep vein thrombosis, prostate cancer, and chronic low back pain, who experienced persistent right-sided back and hip pain despite multiple surgeries, including bilateral sacroiliac joint (SIJ) fusion. Conservative treatments provided only partial relief. A diagnostic SIJ injection with bupivacaine confirmed SIJD as the primary pain source, leading to a radiofrequency ablation (RFA) procedure. The SIJ RFA provided a significant pain reduction.

The treatment algorithm for SIJD prioritizes conservative approaches such as nonsteroidal anti-inflammatory drugs, physical therapy, and SI belts. When these fail, interventional techniques, including joint injections and RFA, are considered before surgery. This case highlights the importance of reassessing SIJD treatment even after SIJ fusion, demonstrating that interventional strategies like repeat RFA can provide meaningful pain relief in select patients.

Effective management of SIJD requires a stepwise approach, with diagnostic injections playing a key role in confirming pain sources. This case underscores the need for individualized treatment strategies, particularly for patients with persistent pain despite prior surgical interventions.

## Introduction

Sacroiliac joint dysfunction (SIJD) contributes to around 15 to 30% of reported cases of lower back discomfort in the general population [1], and this figure increases to approximately 43% in individuals who have previously undergone lumbosacral fusion surgery [2]. The prevalence estimates can vary widely depending on the clinical population and diagnostic approach, with some specialty clinics reporting substantially higher rates. Various factors can trigger sacroiliac joint (SIJ) pain, including trauma, pregnancy, stress, history of lumbar fusion surgery, and the use of bone grafts sourced from areas near the SIJ [3]. The presentation of SIJD symptoms can often resemble other types of lower back pain, emphasizing the importance of accurate diagnosis and effective treatment. A comprehensive assessment relies heavily on the patient’s history and thorough physical examination [4].

Pain arising from the SIJ area typically manifests as discomfort below the beltline, with potential radiation towards the groin or lower extremities. In some cases, patients might report pain radiating below the knee within the L5-S1 dermatome region. Additionally, common symptoms cited by patients include pain during sitting, lying on the affected side, or prolonged walking. To diagnose SIJD, various provocative tests such as the thigh thrust test, Flexion, Abduction and External Rotation (FABER) test, compression test, Gaenslen’s test, sacral thrust test, distraction test, and the Fortin finger test are employed.

A definitive gold standard physical examination for SIJD remains elusive. Research demonstrates that a combination of provocative tests yields superior results compared to individual tests. Positive outcomes are more prevalent when three or more tests are employed [5]. Notably, the combination of FABER and thigh thrust tests demonstrates the highest accuracy, closely followed by the combination of FABER and Gaenslen's test [5]. In terms of imaging techniques, MRI is the most sensitive method for diagnosing SIJD, capable of revealing bone marrow edema and inflammation surrounding the joint [6].

The key diagnostic confirmation for SIJD is the SIJ diagnostic block. A positive indication for the source of SIJ pain is considered when this procedure results in a 50% to 75% reduction of pain [7].

## Case presentation

**Figure 1:**
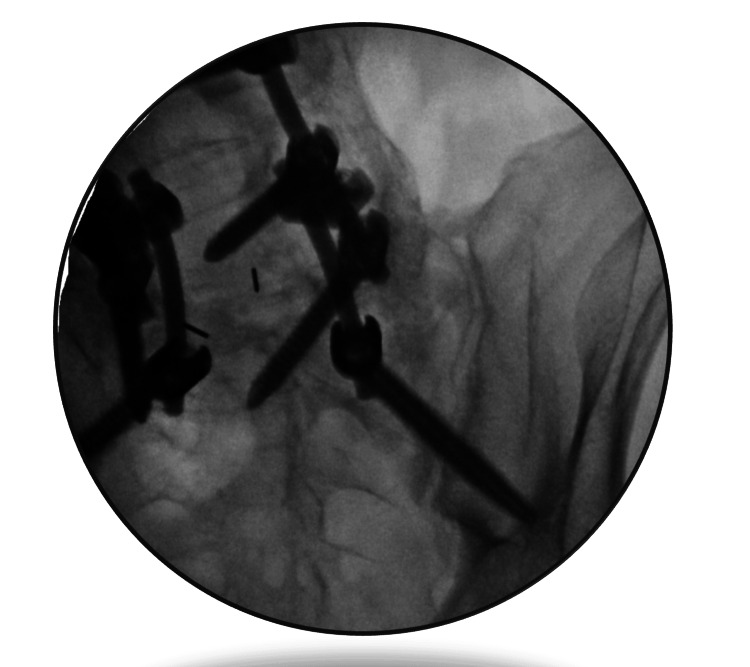
Sacroiliac joint fluoroscopy, showing hardware

A 67-year-old man with a medical history that includes sarcoidosis, deep vein thrombosis (DVT) on Xarelto, prostate cancer, and chronic low back pain was referred to our clinic for an assessment of persistent pain in the right back and hip. He had undergone several surgeries in the past, including L4-S1 foraminotomy, L3-S1 instrumentation, and bilateral open SIJ fusion (Figure 1). Despite some initial relief following these surgeries, his pain progressively worsened over time.

Upon examination, the patient reported experiencing varying levels of pain in his right gluteal region, particularly aggravated when lying down or sitting. He had previously attempted physical therapy and continued home exercises, as well as using an SI belt, which provided partial relief. His current medication regimen included tramadol, gabapentin, baclofen, cyclobenzaprine, and lidocaine patches. Previous interventions consisted of a right hip bursa injection and a right SIJ injection, resulting in partial relief.

Imaging studies showed bilateral SI fusion, multilevel lumbosacral laminectomy and fusion, and narrowing of both SIJs with more pronounced sclerosis on the right side. Limited osseous fusion was observed along the superior right SIJ. Considering the inadequate response to conservative treatments, a diagnostic right SIJ injection was performed to address the patient's persistent pain.

The diagnostic procedure involved a right SIJ injection with 0.25% bupivacaine, which yielded significant pain relief, more than 80% from the baseline pain. Subsequently, the patient was scheduled for a radiofrequency ablation (RFA) procedure to the right SIJ. During the pulsed RFA, three sites were targeted. After confirming the safe placement of the RFA needles through motor and sensory testing, pulsed RFA was performed at 42°C for 120 seconds to target the SIJ region, which provided a partial pain relief afterward. The patient expressed a willingness to undergo another interventional procedure; however, he developed new-onset bradycardia, necessitating evaluation and clearance from his cardiologist prior to proceeding.

**Figure 2:**
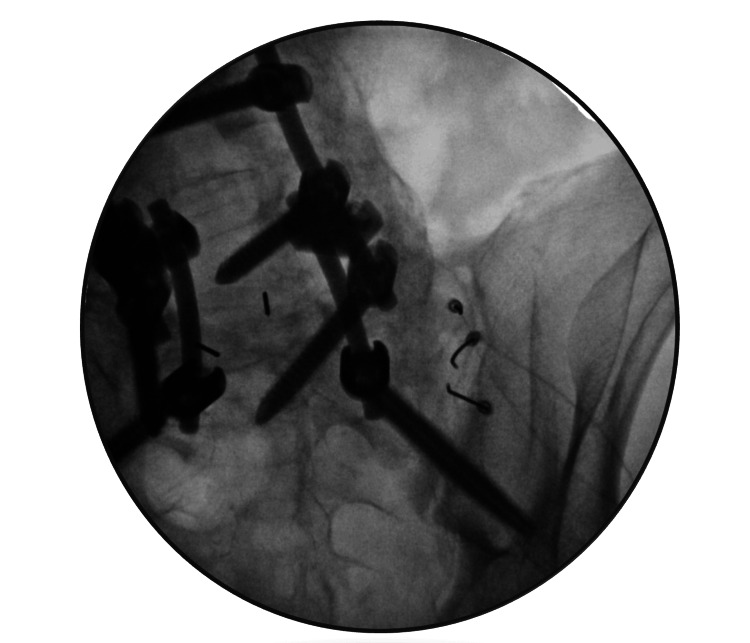
Sacroiliac joint fluoroscopy, showing RFA needle placement RFA: Radiofrequency ablation

Following the required evaluation and cardiologist clearance, a repeat thermal RFA procedure targeting the same three sites in the right SIJ was performed (Figure 2). After confirming the safe placement of the RFA under fluoroscopy with motor and sensory testing, thermal RFA was performed at 80°C for 90 seconds to target the right SIJ. Afterward, the patient reported notable improvement in his pain levels.

## Discussion

After confirming the SIJ as the origin of lower back pain, initial efforts focus on employing conservative treatments for symptomatic patients before considering more invasive options. Conservative measures include nonsteroidal anti-inflammatory drugs (NSAIDs), physical therapy, pelvic belts, and chiropractic manipulations. Should these conservative strategies prove ineffective, more advanced interventions such as extra- and intra-articular injections, prolotherapy, pulsed radiofrequency, radiofrequency denervation, and surgery can be explored as potential treatments for SIJD [8].

Although the precise innervation of the SIJ remains incompletely elucidated, the prevailing understanding suggests a significant contribution from the dorsal rami of L4 to S3 in terms of sensory innervation. It is important to note that there is no standardized procedure established for performing SIJ RFA.

When preceding interventions fail to alleviate pain, SIJ fusion surgery becomes a viable option. In the treatment algorithm for SIJD, if a positive response is obtained from a diagnostic block in patients with persistent pain, the subsequent step often involves RFA. If repeat interventions do not yield the desired relief, consideration shifts toward SIJ fusion surgery [8].

In situations where patients continue to experience intolerable pain postoperatively despite undergoing conservative treatments, options may include revisiting the SIJD treatment protocol. This was the approach taken in the specific case mentioned, where the patient had previously undergone SIJ fusion. By reevaluating the patient's condition within the framework of the SIJD treatment algorithm, pain relief was successfully achieved, highlighting the adaptability and potential efficacy of this approach.

## Conclusions

SIJD is a significant contributor to lower back pain, particularly in individuals with a history of lumbosacral fusion surgery. Given its overlapping symptomatology with other spinal disorders, accurate diagnosis is crucial and relies on a combination of clinical history, provocative tests, imaging modalities, and diagnostic blocks. While conservative treatments such as NSAIDs, physical therapy, and SI belts are the first line of management, interventional procedures, including injections and RFA, offer effective alternatives when conservative measures fail.

The presented case underscores the importance of a systematic and patient-centered approach in managing SIJD. Despite prior surgical interventions, the patient continued to experience significant pain, necessitating further evaluation and targeted interventional strategies. The successful outcome following repeat RFA highlights the role of reassessment and adaptability in the SIJD treatment algorithm. This case reinforces the need for individualized treatment plans and suggests that even in patients with previous SI joint fusion, interventional pain management can provide meaningful relief. However, as a single case report, these findings are inherently limited and should be interpreted with caution. Future research should aim to refine diagnostic protocols and optimize interventional strategies to enhance patient outcomes in SIJD management.
